# Bone Changes During Growth in Patients with Osteogenesis Imperfecta

**DOI:** 10.3390/jcm14051764

**Published:** 2025-03-06

**Authors:** Laura Burgueño-Torres, Lara García-Boedo, Manuel Joaquín de Nova-García

**Affiliations:** Dental Clinical Specialties Department, Faculty of Dentistry, Complutense University of Madrid, 28040 Madrid, Spain; laraga06@ucm.es (L.G.-B.); denova@ucm.es (M.J.d.N.-G.)

**Keywords:** osteogenesis imperfecta, biphosphonates, orthopantomography, panoramic index, pediatric patient

## Abstract

**Background/Objectives**: Osteogenesis Imperfecta (OI) is a congenital disorder, in which the production of collagen, mainly type I, is altered, leading to a decrease in bone mineral density, increasing the risk of fracture with minimal trauma. Several studies have analyzed bone mineral density in osteoporotic patients based on linear measurements such as radiomorphometric indices measured with panoramic radiographs, although few studies have investigated bone trabeculation in children diagnosed with OI. Therefore, the aim of the present investigation was to analyze the dental panoramic indices in panoramic radiographs in the cortical and trabeculated bone of children with OI. **Methods**: Thus, 66 pediatric patients diagnosed with OI under antiresorptive treatment were compared with a sample of controls matched for sex and age. Using Image J software (version: 1.54d), three radiomorphometric indices were analyzed in orthopantomographies of the study and control groups, evaluating the influence of disease severity as well as the type of antiresorptive treatment administered. **Results**: Patients with OI had a higher presence of type C2 and C3 MCI (mandibular cortical index) than their matched controls (*p* < 0.05), although no differences were found for the visual estimation of cortical width (SVE) and mandibular cortical width (MCW). Treatment with zoledronic acid was associated with a higher number of cases of type C1 MCI, in terms of sample description, while patients treated with a combination of pamidronate and zoledronic acid had a higher rate of type C1 and C2 MCI, with no statistical differences. **Conclusions**: In the overall sample, most patients showed a thin SVE index (59.1%), a C2 or C1 type MCI (46.2% and 42.4%) and an MCW of 2.9 mm. Differences in bone mineral density were also observed throughout growth and the different antiresorptive treatments. Zoledronic acid has been associated with a higher percentage of C1 and C3 ICM, and pamidronate alone or in combination is associated with a C1 and C2 MCI index.

## 1. Introduction

Osteogenesis Imperfecta is a disease characterized by an alteration in collagen synthesis that directly affects bone metabolism, characterized by a decrease in bone mineral density in these patients [1,2,3]. It is a genetically heterogeneous skeletal dysplasia, affecting approximately 1 in 10,000–20,000 births. This estimate is a lower limit, as milder forms of the disease frequently go undiagnosed [4,5]. At the oral and craniofacial level, they will present a series of alterations that are classic and are directly related to bone and skeletal alterations. Patients with OI tend to present Class III skeletal malocclusions, with crossbite due to maxillary hypoplasia, posterior open bite and characteristic dental alterations, such as dentinogenesis imperfecta [1,2,6,7].

Over the last few years, several investigations have been carried out to analyze the usefulness of panoramic radiographs as a way of assessing the osteoporotic status of these patients, analyzing some radiomorphometric indices and evaluating their potential indicator of a decreased bone mineral density value. The existing literature contains information on these indices, such as the Goniac Index [8,9], the Antegonial Index [10,11], the resorption index [12] or the Panoramic Mandibular Index, initially defined by Benson et al. [13,14] and adapted by Taguchi [15] as a measure of mandibular cortical thickness. Klemetti et al., in 1993 [8], also defined the mandibular cortical index by differentiating three categories according to its thickness. Camargo et al. [16] found evidence of the applicability of the method of mandibular cortical thickness evaluated on panoramic radiography to determine the degree of bone mineral density in osteoporotic patients, which was also supported by Pacheco-Pereira et al. (2018) [17] and Rocha et al. (2019) [18].

However, there are few studies in the literature evaluating the applicability of these indices when analyzing bone mineral density in pediatric patients with OI. Apolinario et al. [19] in 2015 published the first study evaluating the effectiveness of panoramic radiographs as a method of assessing bone mineral density in these patients.

Currently, bisphosphonate therapy is the gold standard in patients with OI, increasing their bone mineral density and reducing the risk of fractures [20,21,22,23]. Bisphosphonates mainly improve bone mass by inhibiting osteoclasts, which leads to the inhibition of bone resorption, especially on the inner surfaces of the bones. While bisphosphonates do not improve the quality of abnormal bone in OI, they do increase overall bone mineral density, thus improving mechanical strength. The greatest increase in bone density occurs during the first 2 to 4 years of treatment [24,25,26,27]. Pediatric patients with OI have periodic medical controls, with the aim of monitoring the effectiveness of the antiresorptive treatments administered to them by performing clinical, radiological (densitometry) and histological (detection of bone remodeling markers in blood) examinations [28]. In addition, panoramic radiography is used as a routine method of diagnosis in these patients for the treatment of their oral and dental alterations, especially during the mixed dentition phase. This makes it essential to evaluate the capacity of this diagnostic method as an indicator of bone mineral density in these patients, which would make it possible to determine the progress of the treatments. This research is based on the needs for a protocolized study that examines the changes in the cortical and trabecular bone of the jaws measured in panoramic radiographs in children with OI, who have received different therapeutic protocols with antiresorptive drugs.

Therefore, the objectives of the present study were to analyze the radiomorphometric indices in panoramic radiographs of children with OI treated with bisphosphonates and to compare them with a sample of healthy control children.

## 2. Materials and Methods

### 2.1. Ethical Aspects

The present study was approved by the Ethics Committee for Research with Drugs of the Clinic Hospital San Carlos (C.I. 21/316-O_M_OD).

All subjects included signed informed consent to participate in the research. In compliance with current legislation, and in accordance with Organic Law 15/1999 on Personal Data Protection, the confidentiality of each of the subjects included in the study was respected by coding their data by consecutive numbering as they joined the study.

### 2.2. Study Design

A cross-sectional, retrospective, analytical and observational study was designed, in which measurements were made on panoramic radiographs of children with OI compared with a group of healthy subjects matched for age and sex.

Three radiomorphometric indices were evaluated to assess differences in bone mineral density between the two groups.

Data collection was conducted between October 2021 and October 2023.

### 2.3. Study and Control Samples

The study sample consisted of children with OI who attended the “Master’s Degree in Pediatric Dentistry” and the “Specialist in Integrated Dental Care for Children with Special Needs” of the Faculty of Dentistry of the Complutense University of Madrid.

To be included in the investigation, all the subjects had to be diagnosed with OI and be treated or not with bisphosphonates, European and Spanish origin, between 5 and 23 years old, have a previous good-quality panoramic radiograph, and obtained informed consent from the patient or parents/guardians. Those subjects with disorders that directly affect bone mineral density (osteopenia, osteoporosis, Paget’s disease, etc.), poor-quality panoramic records and not having the informed consent of the patient/parents/guardians were excluded.

Following these criteria, the study sample consisted of 66 subjects (28 boys and 38 girls), with an age range between 5 and 23 years. The mean age was 9.88 ± 3.92 years (median 9). The central 50% was between 7 and 11 years of age. The median age was used to dichotomize the sample into two groups for comparison (older and younger than 9 years).

All patients in the study sample were categorized according to OI type diagnosed: OI I (45.5%; *n* = 30), OI II (1.5%; *n* = 1), OI III (21.2%; *n* = 14), OI IV (22.7%; *n* = 15), OI V (9.1%; *n* = 6). In order to improve the statistical and comparative analysis of the study sample, the patients were classified according to the severity of the disease, dividing the sample into three groups:

- Mild OI: 45.5%, *n* = 30
- Moderate OI: 31.8%, *n* = 21
- Severe OI: 22.7%, *n* = 15

Approximately a quarter of the study sample (27.27%; 18 patients) did not have any type of antiresorptive therapy administered, while the remaining 72.72% (48 patients) had different bisphosphonate therapies.

The control sample group consisted of healthy subjects matched for age and sex with the study sample. All had to have panoramic records of adequate quality and signed informed consent to participate in this study. This sample also consisted of 66 patients (28 boys and 38 girls).

### 2.4. Systematics of the Investigation

**Figure 1:**
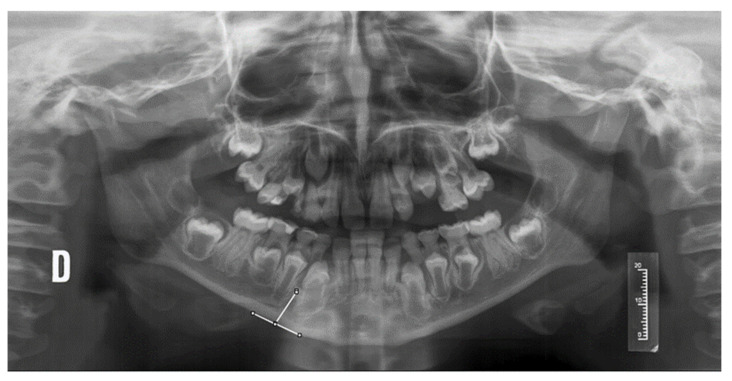
Measurement of the MCW index in an orthopantomography of a patient with OI. D: right side of the patient.

The radiomorphometric indices analyzed were as follows:

- Mandibular Cortical Index (MCI) classified into three categories: C1 (normal cortex with continuous and flat endosteal margin), C2 (endosteal cortical sediments on one or both mandibular sides, or semilunar deficiencies in the endosteal margin), C3 (porous cortex with endosteal defects).
- Visual estimation of cortical width (SVE), classified as normal (>3 mm) or thin (<3 mm).
- Mandibular cortical width (MCW), measured from a tangential to the mandibular border and another parallel to the ascending ramus, obtaining a tangent through the mental foramen (Figure 1).

All measurements were carried out by two double-blinded examiners. The first examiner measured the entire sample, carrying out a second measurement of 20% of the sample with a time interval of 30 days, while the second examiner assessed 20% of the sample. Intra-examiner reliability and inter-examiner agreement were evaluated.

### 2.5. Statistical Analysis

Statistical analysis of the data was performed with SPSS Statistics 25.0 for Windows. The mean difference test (Student’s *t*-test) was used for normal variables and a non-parametric alternative (Wilcoxon test) for variables that were not distributed according to statistical normality. For categorical variables in matched groups, McNemar’s test was used. Inter-observer reliability was calculated using correlation coefficients, scatter plots and a contrast test on the difference = 0.

## 3. Results

A total of 66 subjects with OI were included in the study sample, with a female predominance (57.6%), although there were no statistical differences in the group (Table 1). The mean age was 9.88 years (the central 50% of the sample was between 7 and 11 years of age), which was used as a criterion to classify the patients according to age group, taking this fact into account when analyzing the cycles of bisphosphonates administered. Almost half of the sample (45.5%) was made up of patients with type I OI, the rest being divided into patients with type IV and V OI (31.8%) and type III (21.2%).

### 3.1. BMD in the Overall Sample

**Figure 2:**
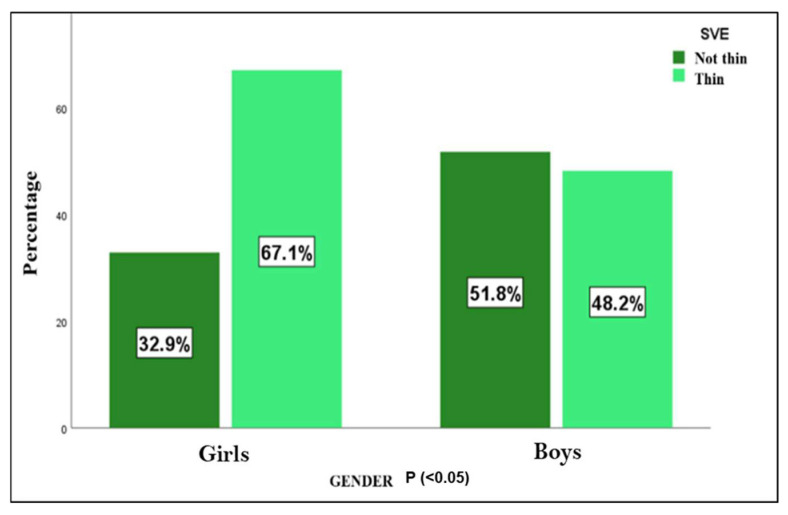
SVE—visual estimation of cortical width. Differences between both sexes (*n* = 132).

When analyzing the SVE index, it was observed that in most cases (59.1%; *n* = 78), this width was thin, with a higher frequency in the group of girls (*p* < 0.05), as shown in Figure 2. In addition, thin SVE was much more frequent in younger subjects (≤9 years), finding this difference statistically significant (*p* < 0.001), thus finding solid evidence that age is a differential factor on SVE.

**Figure 3:**
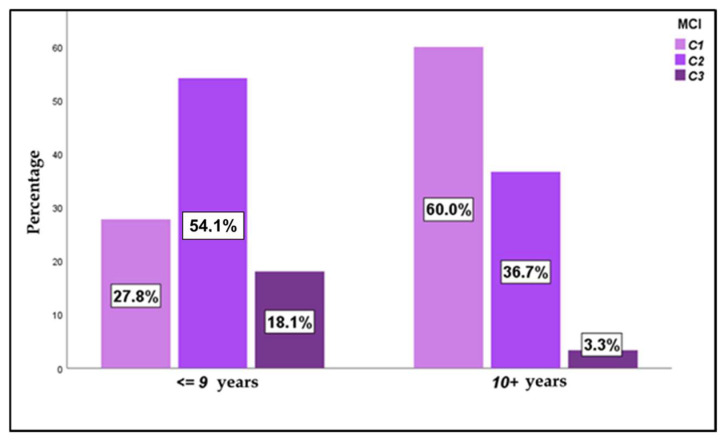
MCI—mandibular cortical index, according to age in the overall sample (*n* = 132).

When studying the mandibular cortical index (MCI), most of the subjects presented C1 and C2 grades, with a slightly higher presence of C2 (46.2% of C2 versus 42.4% of C1). Only 11.4% of the patients presented a C3 score, with no differences found among both sexes. Analyzing the age factor (Figure 3), statistically significant differences were observed (*p* < 0.000), with a much higher frequency of C1 among those older than 9 years (60.0% in those older than 9 years and 27.8% in those younger than 9 years).

Upon analyzing the values obtained in the MCW index in the overall sample, significant differences were observed with respect to sex, with higher indices in males (3.19 vs. 2.68 in females; *p* < 0.01). Statistically significant differences were also obtained with respect to patient age (*p* < 0.01), with higher indices observed in older patients (3.45 vs. 2.43).

### 3.2. Influence of the Presence of OI on BMD

However, when studying the influence of OI on SVE, we observed that the proportion of subjects with a cortical width considered thin was similar in both groups (57.6% in the study sample and 60.6% in the control sample), without a statistical difference (*p* > 0.05) and with a near-zero effect size. Therefore, there is no statistical evidence indicating differences in SVE between the OI cases and the sex- and age-matched control group (Table 2).

When analyzing the differences between the study and control samples, the MCI index showed statistically significant differences between both groups (*p* < 0.001), with a large effect size (R^2^: 22%), indicating a greater statistical strength in the differences found (Table 1). The data showed that in the OI group, there were fewer C1 cases (22.7% in the study sample vs. 62.1% in the control sample), more C2 cases (54.5% in the study sample vs. 37.9% in the control sample) and considerably more C3 cases (22.7% in the study sample vs. 0% in the control sample).

When analyzing the influence of OI on the MCW index, we found very similar mean values in both groups, with a difference so small that it resulted in a null effect size and not statistically significant values, finding very similar MCW values between patients in the study and control groups.

About one-third of the patients included in the study sample (*n* = 18) were not receiving any type of bisphosphonate treatment; therefore, following the premise of objectifying the influence of the disease on the mandibular cortex, the values obtained in this group were compared with their matched controls.

Possibly due to the small sample size, no significant differences were found in either SVE or MCW. However, when analyzing the results in MCI, a high significance was found, observing that in the group of cases with OI, C2 and C3 were more frequent, while C1 was much less frequent (Table 3). This result should be taken with caution, given the small sample size, which is a limitation in this research; nevertheless, most of the patients included were treated with bisphosphonates.

### 3.3. Influence of Medication on BMD

All the patients provided detailed and updated medical reports, from which information was obtained on the different therapies administered:

- 27.3% (*n* = 18) received no treatment.
- 15.2% received treatment with pamidronate (*n* = 10).
- 16.7% received treatment with zoledronic (*n* = 11).
- 40.9% were treated with both medications (*n* = 27).

Based on the protocol established for the administration of bisphosphonates for Osteogenesis Imperfecta, the dose used in the case of Pamidronate is 1 mg/kg/dose in cycles of 3 days every 3 months and Zoledronic Acid 0.05 mg/kg/dose every 6 months.

According to the results obtained in the present investigation, no statistical differences were obtained for any of the indices analyzed. However, a moderate effect size was obtained when analyzing the MCI variable, which is an indicator of a possible relationship. Based on the data, there seems to be a link between the use of pamidronate (or its combination with zoledronate) and C2 type MCI. However, the small sample size in this case means that we should take this association with caution until the sample is increased (Table 4). The administration of zoledronic acid was associated with a higher number of C1 MCI cases, both alone and in association with pamidronate, with no statistically significant differences, possibly due to the small sample size, but with a moderate effect size, which may indicate a possible association.

Therefore, 27 patients in the study sample had the combined administration of pamidronate and zoledronic acid; of these, 22 showed C1 and C2 MCI. In the case of pamidronate, 10 patients in the study sample had the single administration of this drug, with 8 of them showing a C2 MCI.

In order to complement the analysis, the relationship of the cumulative dose of bisphosphonates with each of the studied cortex indices was analyzed, and no statistical differences were found in any of them. As in the previous analysis, in this case, a moderate-mild effect size was also found for the MCI index, but no positive statistical relationship could be affirmed.

### 3.4. Influence of the Severity of OI with BMD

In light of the data obtained, the mean values of MCW and SVE were very similar for the three degrees of severity of the disease. Only in the case of MCI were there some differences that could not be taken into account in a significant way. In this regard, the results of this research point to a higher presence of C1 in severe OI, C2 in mild/moderate OI and C3 in mild OI (Table 5).

## 4. Discussion

Knowledge of changes in BMD becomes essential in multiple diseases, and its monitoring in the evolution of various pathologies justifies finding increasingly non-invasive methods that help evaluate these pattern changes. This fact becomes even more relevant when we are talking about developing patients, for whom panoramic radiography is a routine diagnostic tool.

This methodology based on the analysis of orthopantomographies has not only been used in the diagnosis of osteoporotic patients and patients with bone-related diseases such as OI but also in studies of patients with bruxism compared to control groups [29] and studies of skeletal malocclusions treated orthopedically with functional appliances and untreated [30]. In addition, it has been applied in cases of obstructive sleep apnea [19], instances of agenesis of mandibular second premolars [31], and in studies related to associations regarding bone alterations with certain control groups [9,10,12,14,15,16,31,32].

Klemetti et al. in 1993 pioneered the analysis of radiomorphometric indices to correlate mandibular BMD with femoral and lumbar BMD in an adult population [8]. In subsequent years, many authors used these indices for the analysis of BMD at different levels [9,10,11,12,13,14,15,16,19,30,31,32,33,34,35,36,37,38,39,40].

Based on the existing literature, we selected the MCW, MCI and SVE as the indices to be studied, due to their calculation ease and accuracy.

According to Apolinario et al. (2015), differences in the MCW, MCI and SVE parameters are observed between men and women [19]. The present study finds differences only in MCW with respect to sex, unlike the rest of the indices analyzed, which showed no differences between boys and girls, with higher values in male patients and lower values in females. This, in fact, is related to the greater bone density in males in general, which is directly related to MCW.

When changes in the different indices were analyzed with respect to growth, the present study reports a relationship between age and the MCW index, observing that cortical width increases with growth due to greater bone mineral density, finding statistically significant differences. According to the results presented, the SVE appears thin in children ≤9 years of age, which is explained taking into account that the SVE increases with age due to the higher bone mineral density that occurs with growth. The MCI index tends to be more frequent with type C1 at older ages. According to Apolinario et al. [19], the patients in the control sample presented variations in bone mineral density by age group, with similar values in the groups of children aged 8 to 10 years and 11 to 14 years. Prado et al. [41], in their 2024 investigation, also found significant differences in the MCI index in the study group with respect to the control sample, although, in this case, the study sample is very limited (20 patients), with a 1:2 ratio with respect to the healthy controls, and the results should be taken with caution. According to these authors, 80% of patients showed C2 and C3 MCI indices versus 90% of controls showing C1 MCI indices. However, in this investigation, the samples were not matched and neither was the influence of disease severity nor of the treatment administered.

In children with OI, authors such as Apolinario et al. [19] also observed variability according to age, with exceptions in the groups of 5 to 7 years old and 8 to 10 years old, as well as between the groups of 10 to 14 years old and 15 to 19 years old. In both groups, the MCW value increases with age, being lower in patients with type I and III OI. According to our results, patients with OI present a lower BMD than healthy patients, with more prevalence of C2 and C3 indices, where the bone is more porous with remnants of endosteal debris and semilunar defects in the lower mandibular cortex, with osteopenia and weakness in the bone structure. However, the control group presents more prevalence of C1 index, where the margin of the lower mandibular cortex is uniform, characteristic of healthy bone tissue.

In a later investigation, the same authors concluded that there were no differences in MCI and SVE values between age groups, in contrast to the results presented in this research, in which a greater coincidence was found with type C1 MCI and SVE at older ages [42].

Likewise, in this research, it was observed that MCI C1 was more frequent in those older than 9 years, which is relevant when considering the administration of bisphosphonates in elderly patients. The results showed an association between SVE and MCW measurements, as well as between MCI and MCW. Low MCW values were associated with C3 type MCI measurements and a thin lower mandibular cortex in patients with OI, whereas in healthy patients, low MCW was associated with C2 MCI.

In the study sample presented, different antiresorptive therapies were administered, either pamidronate or zoledronic acid alone or a combination of both. In addition, the dose of drug administered was taken into account, as well as the cumulative dose over time. The results show that there is no correlation between the administration of pamidronate or zoledronate in the variables MCI, MCW and SVE. However, in SVE, although it did not reach statistical significance, a small correlation was observed between SVE and the administration of zoledronic acid, in which for greater drug administration, a non-thin SVE was observed.

## 5. Conclusions

The results of the study sample show that the majority have a thin SVE (59.1%), an MCI of type C2 (46.2%) or C1 (42.4%) and an MCW of 2.9 mm. In addition, males older than 9 years have lower osteoporotic indices. Statistically significant differences in measurements were found between the OI groups and their healthy controls, with fewer cases of C1 MCI in the OI group and more cases of C2 and C3. However, no statistical differences were observed in SVE and MCW measurements.

Treatment with pamidronate alone or in combination with zoledronic acid is associated with a C1 and C2 type mandibular cortical index (MCI), especially in mild and moderate forms of OI. The use of zoledronic acid alone was associated with a higher number of cases of type C1 MCI in the more severe forms and C3 in mild OI.

## Figures and Tables

**Table 1:** Descriptive analysis of the sample.

	Overall Sample (*n* = 132)	OI Sample (*n* = 66)	Control Sample (*n* = 66)
***AGE***			
Mean (SD)	9.88 (±3.92)	9.88 (±3.92)	9.88 (±3.92)
Median	9.00 (5–23)	9.00 (5–23)	9.00 (5–23)
***SEX***			
Girls	57.6% (76)	57.6% (38)	57.6% (38)
Boys	42.4% (56)	42.4% (28)	42.4% (28)
***OI TYPE***			
OI I	-	45.5% (30)	-
OI II	-	1.5% (1)	-
OI III	-	21.2% (14)	-
OI IV	-	22.7% (15)	-
OI V	-	9.1% (6)	-

**Table 2:** Radiomorphometric indices, compared between study and control sample (*n* = 132).

Variable	Rate (Frequency)		Contrast Test (Mc Nemar)		Effect Size R^2^
	OI (*n*)	Control (*n*)	Statistical	*p*-Value	
**SVE**			0.45	0.824	0.009
Thin	57.6% (38)	60.6% (40)			
Not Thin	42.4% (28)	39.4% (26)			
**MCI**			5.06	0.000	0.220
C1	22.7% (15)	62.1% (41)			
C2	54.5% (36)	37.9% (25)			
C3	22.7% (15)	0.0 %			
**MCW**	2.89 (±1.21)	2.91 (±0.93)	t = 0.09	0.927	0.000

**Table 3:** Radiomorphometric indices, compared between matched groups (*n* = 18).

Variable	Rate (Frequency)		Contrast Test (Mc Nemar)		Effect Size R^2^
	OI	Control Group	Statistical	*p*-Value	
**SVE**			0.08	0.990	0.000
Thin	55.6%	44.4%			
Not Thin	50.0%	50.0%			
**MCI**			2.65	0.008	0.021
C1	16.7%	55.6%			
C2	61.1%	44.4%			
C3	22.2%	0.0%			
**MCW**	3.28 (±1.35)	3.09 (±0.79)	Z_W_ = 0.76	0.468	0.021

**Table 4:** Radiomorphometric indices according to pharmacological treatment (*n* = 48).

Variable	Rate (Frequency)			Contrast Test (Chi-Square)		Size Effect R^2^
	Pamidronate and Zoledronic Acid (*n* = 27)	Pamidronate (*n* = 10)	Zoledronic Acid (*n* = 11)	Statistical	*p*-Value	
**SVE**				0.22	0.894	0.005
Thin	55.6% (15)	60.0% (6)	63.6% (7)			
Not Thin	44.4% (12)	40.0% (4)	36.4% (4)			
**MCI**				7.34	0.119	0.077
C1	29.6% (8)	10.0% (1)	27.3% (3)			
C2	51.9% (14)	80.0% (8)	27.3% (3)			
C3	18.5% (5)	10.0% (1)	45.5% (5)			
**MCW**	2.93 (±1.16)	2.52 (±0.80)	2.52 (±1.34)	F = 0.76	0.476	0.032

**Table 5:** Radiomorphometric indices according to the type of OI (*n* = 66).

Variable	Rate (Frequency)			Contrast Test (Chi-Cuadrado)		Size Effect R^2^
	Mild OI (*n* = 30)	Moderate OI (*n* = 21)	Severe OI (*n* = 15)	Statistical	*p*-Value	
**SVE**				0.18	0.912	0.002
Thin	60.0% (18)	57.1% (12)	53.3% (8)			
Not thin	40.0% (12)	42.9% (9)	46.7% (7)			
**MCI**				5.37	0.252	0.041
C1	13.3% (4)	23.8% (5)	40.0% (6)			
C2	56.7% (17)	61.9% (13)	40.0% (6)			
C3	30.0% (9)	14.3% (3)	20.0% (3)			
**MCW**	2.83 (±1.32)	2.88 (±0.98)	3.03 (±1.35)	F = 0.12	0.885	0.004

## Data Availability

The data presented in this study are available on request from the corresponding author upon reasonable request. The data are not publicly available due to privacy restrictions.

## Abbreviations

The following abbreviations are used in this manuscript:

OI	Osteogenesis Imperfecta
BMD	Bone Mineral Density
SVE	Visual estimation of cortical width
MCI	Mandibular Cortical Index
MCW	Mandibular Cortical Width
